# Souvenaid in the management of mild cognitive impairment: an expert consensus opinion

**DOI:** 10.1186/s13195-019-0528-6

**Published:** 2019-08-17

**Authors:** Jeffrey Cummings, Peter Passmore, Bernadette McGuinness, Vincent Mok, Christopher Chen, Sebastiaan Engelborghs, Michael Woodward, Sagrario Manzano, Guillermo Garcia-Ribas, Stefano Cappa, Paulo Bertolucci, Leung-Wing Chu

**Affiliations:** 1 Department of Brain Health, School of Integrated Health Sciences, UNLV; Cleveland Clinic Lou Ruvo Center for Brain Health, Las Vegas, NV USA; 2 Centre for Public Health, Institute of Clinical Sciences, Queens University Belfast, Belfast, UK; 3 Therese Pei Fong Chow Research Center for Prevention of Dementia, Gerald Choa Neuroscience Centre, Lui Che Woo Institute of Innovative Medicine, Department of Medicine and Therapeutics, The Chinese University of Hong Kong, Hong Kong Special Administrative Region, China; 4 Departments of Pharmacology and Psychological Medicine, Yong Loo Lin School of Medicine, National University of Singapore, Memory Aging and Cognition Centre, National University Health System, Singapore, Singapore; 5 Reference Centre for Biological Markers of Dementia (BIODEM), University of Antwerp, Antwerp, Belgium; 6 Department of Neurology, Centre for Neurosciences, UZ Brussel, Vrije Universiteit Brussel (VUB), Brussels, Belgium; 7 Department of Medicine, The University of Melbourne, Austin Health, Melbourne, Victoria Australia; 8 Neurology Department, Infanta Leonor Hospital, Madrid, Spain; 9 Servicio de Neurología, Hospital Universitario Ramón y Cajal, Madrid, Spain; 10 University School for Advanced Studies IUSS, Pavia and IRCCS Istituto Centro, S. Giovanni di Dio, Brescia, Italy; 11 Service of Cognitive and Behavioral Neurology, Department of Neurology and Neurosurgery, Federal University of São Paulo, São Paulo, Brazil; 12 Department of Medicine, The University of Hong Kong and Hong Kong Brain Memory Centre, Hong Kong Special Administrative Region, China

**Keywords:** Mild cognitive impairment, Prodromal Alzheimer’s disease, Nutrient, Diet, Souvenaid, Memory, Cognition

## Abstract

**Background:**

Mild cognitive impairment (MCI) among an aging global population is a growing challenge for healthcare providers and payers. In many cases, MCI is an ominous portent for dementia. Early and accurate diagnosis of MCI provides a window of opportunity to improve the outcomes using a personalized care plan including lifestyle modifications to reduce the impact of modifiable risk factors (for example, blood pressure control and increased physical activity), cognitive training, dietary advice, and nutritional support. Souvenaid is a once-daily drink containing a mixture of precursors and cofactors (long-chain omega-3 fatty acids, uridine, choline, B vitamins, vitamin C, vitamin E, and selenium), which was developed to support the formation and function of neuronal membranes and synapses. Healthcare providers, patients, and carers require expert advice about the use of Souvenaid.

**Methods:**

An international panel of experts was convened to review the evidence and to make recommendations about the diagnosis and management of MCI, identification of candidates for Souvenaid, and use of Souvenaid in real-world practice. This article provides a summary of the expert opinions and makes recommendations for clinical practice and future research.

**Summary of opinion:**

Early diagnosis of MCI requires the use of suitable neuropsychological tests combined with a careful clinical history. A multimodal approach is recommended; dietary and nutritional interventions should be considered alongside individualized lifestyle modifications. Although single-agent nutritional supplements have failed to produce cognitive benefits for patients with MCI, a broader nutritional approach warrants consideration. Evidence from randomized controlled trials suggests that Souvenaid should be considered as an option for some patients with early Alzheimer’s disease (AD), including those with MCI due to AD (prodromal AD).

**Conclusion:**

Early and accurate diagnosis of MCI provides a window of opportunity to improve the outcomes using a multimodal management approach including lifestyle risk factor modification and consideration of the multinutrient Souvenaid.

**Electronic supplementary material:**

The online version of this article (10.1186/s13195-019-0528-6) contains supplementary material, which is available to authorized users.

## Background

Mild cognitive impairment (MCI) is heterogeneous but in approximately 50% of cases represents a transitional state between normal aging and dementia [1, 2]. Early diagnosis of Alzheimer’s disease (AD) in the MCI/prodromal stage presents an opportunity for interventions to improve brain health or cognitive functioning and to manage modifiable risk factors implicated in disease progression [3]. The objectives of this paper are to describe and evaluate the current identification and management of patients with MCI and to assess the role of Souvenaid (a multinutrient product) in the management of the MCI population. Since the term MCI covers a wide range of clinical presentations, an important objective was to examine the potential role of Souvenaid based on the evidence that specifically defined MCI and AD subtypes. In particular, this paper focuses on the management of patients with MCI with underlying AD pathology (e.g., prodromal AD or MCI due to AD).

Diet and nutritional status are recognized as important considerations in healthy brain aging and dementia [4]; however, clinical trial evidence for the effectiveness of single nutritional interventions in MCI remains limited [5]. Randomized clinical trials of Souvenaid have provided evidence for improvement in memory performance in subjects with early Alzheimer’s disease (AD) [6, 7], but not in those with more advanced stages of AD [8]. More recently, European investigators reported encouraging findings from a trial in subjects with prodromal AD (MCI due to AD), which showed that Souvenaid had beneficial effects on cognition and function (Clinical Dementia Rating Scale-Sum of Boxes [CDR-SOB] and AD Composite Score [ADCOMS]) and on hippocampal atrophy rate [9, 10]. These data raise the possibility of considering Souvenaid as a management option for individuals diagnosed with MCI due to AD.

This paper summarizes the key clinical issues relevant to the use of Souvenaid in MCI due to AD and is based on expert insights and consensus opinions provided at a meeting held in July 2018 attended by the authors and sponsored by Nutricia. The participants represented many countries, and the consensus statement presents a global view of MCI management. After the consensus meeting, we searched ClinicalTrials.gov, WHO’s International Clinical Trial Registry Platform, and PubMed to find new clinical trials and publications about nutritional interventions in subjects with MCI, using the specific search terms “Alzheimer’s disease,” “mild cognitive impairment,” “nutrition,” “Souvenaid,” and “Fortasyn Connect.” All authors contributed to the paper and have approved the contents.

Role of the sponsor: Nutricia paid for the meeting room but not travel costs and did not influence the content of the consensus meeting. No Nutricia employees are listed as authors. The authors had full editorial control and made the decision to submit the final manuscript for publication.

## How do physicians identify and diagnose MCI?

Individuals with MCI show a decline in cognitive functioning greater than expected for age and educational background [11]. People with MCI or their partners and other family members become aware and complain about cognitive deficits, and the initial visit to the primary care physician is usually precipitated by such complaints. Amnestic MCI typically affects recent episodic memory function and may impact several other cognitive domains including executive function, language, and visuospatial skills. People with MCI are independent with regard to the activities of daily living.

In community practice, problems with memory commonly raise the physicians’ suspicions of MCI or dementia [3, 12]. Primary care physicians are encouraged to assess the patients complaining of memory loss or to refer these individuals with signs of MCI for specialist memory assessment because of the high risk of progression to dementia, most commonly AD [13]. In general, approximately 50% of MCI are associated with amyloid pathology, and 10–15% per year will transition to dementia of the AD type. The estimated 3-year cumulative incidence of AD-type dementia for individuals presenting with prodromal AD (International Working Group (IWG-2) criteria) is 61% [14]. On the other hand, a systematic review reported reversion rates from MCI to normal cognition of 8% in clinical-based studies and 25% in population-based studies [15].

US and international guidelines recommend specialist assessment using validated tests of memory and cognitive function for individuals with signs and symptoms of memory impairment or when family members or patients express concerns about potential cognitive decline [16–19]. Patient associations also play a key role in increasing awareness and encouraging early diagnosis of MCI.

The success of the strategies to delay the progression of MCI to dementia depends on early and accurate identification of people at risk of AD, including those without any evidence of significant neurodegeneration [20]. Appropriate assessment by the primary care physician including screening for cognitive problems is important to expedite referral of individuals with suspected MCI to specialists, e.g., geriatricians, psychiatrists, or neurologists [21, 22]; however, many patients are not referred [23] and some healthcare organizations may even discourage the use of specialists [24]. Timely referral and diagnosis of MCI can motivate individuals to adhere to potentially beneficial lifestyle changes and treatment interventions [11].

In the early stages, MCI may be differentiated from dementia by a preservation of functional independence and the absence of significant impairment in social or occupational functions [25]; however, the precise boundary remains elusive and there is a seamless progression from MCI to mild dementia. Further research is advocated to improve the accuracy and utility of assessment tools, such as activity of daily living (ADL) scales, and to refine screening tests at each step of the pathway to diagnosis. There is a need to improve early diagnosis of MCI allowing appropriate interventions designed to improve patient or caregiver outcomes [25].

The IWG-2 criteria define, in the context of research, prodromal AD as patients with episodic memory impairment (in most cases) and a positive cerebrospinal fluid amyloid beta (Aβ) and tau biomarker test or a positive amyloid beta-protein (Aβ) positron emission tomography (PET) scan [20, 25, 26]. In the National Institute on Aging-Alzheimer’s Association criteria, among patients meeting the core clinical criteria for MCI due to dementia, the highest likelihood of underlying AD pathology is associated with positive biomarkers for both Aβ and neuronal injury, while an intermediate risk is associated with either a positive biomarker reflecting Aβ deposition with an unavailable biomarker of neuronal injury or a positive biomarker reflecting neuronal injury with an unavailable biomarker of Aβ [19]. Further research is important to improve early diagnosis and to facilitate more individualized management [26].

Individuals with suspected MCI should have a comprehensive medical history and physical examination to distinguish between MCI and normal aging or dementia and to identify individuals with potentially reversible (or irreversible) MCI caused by other underlying conditions [25]. Neuropsychiatric assessment is advocated by some experts because apathy, depression, and agitation are often the early symptoms of MCI [27]. An evaluation may also identify patients in early phases of non-AD conditions such as frontotemporal dementia and dementia with Lewy bodies.

Cognitive function assessments are recommended at baseline and follow-up visits [25, 28, 29]; however, the utility of available tools may be limited because many practitioners lack familiarity with them and most have administration times incompatible with the short visit times of many busy practices. Additional research is needed to harmonize neuropsychological tests for use in different languages [26]. Furthermore, the sensitivity of such instruments to detect MCI may be lower than that to detect dementia [28]. Available screening instruments are not fully appropriate for MCI assessment because they do not allow a precise quantitative definition of domain-specific impairment, for example, in episodic memory, with reference to an age- and education-matched population.

While brief screening tools are appropriate in general practice, at a specialist level, neuropsychological assessment is required. Neuropsychological tests provide valuable objective information; they can reinforce but never supplant clinical judgment and communication with the patient. The patient’s clinical history is required to identify the presence of cognitive complaints, elicited from the individual, family member, or colleague. A detailed clinical history is essential to reflect inter-personal changes, specifically considering the individual’s behavior and social cognition before MCI emerged. Monitoring the changes in cognitive function, memory complaints, functional abilities, and personality over time is essential, and collecting a relevant family history may contribute additional important information.

Expert opinion:

- Early diagnosis of MCI requires the use of suitable neuropsychological tests combined with a careful clinical history. Physicians should explore the clinical history because it provides important information about the changes in individual patients, which may alert them to the emerging cognitive impairment even when an objective screening test is normal.
- Biomarkers may be used to diagnose prodromal AD in patients with MCI.

## Current management of MCI

Expert reports have concluded that some medical, lifestyle, psychosocial, and nutritional interventions may prevent or delay the progression from MCI to dementia [3, 30]; however, evidence is not robust for many interventions, and no significant benefits have been shown with pharmacologic therapies such as cholinesterase inhibitors and memantine [16, 30, 31]. The Lancet Commission on Dementia Prevention suggested that 21.7% of dementia cases progressing from MCI are potentially preventable by eliminating poor diets, diabetes, and neuropsychiatric symptoms [3]. Attention to modifiable risk factors is therefore the first step for patients diagnosed with MCI [3]. Cognitive training, blood pressure management in people with hypertension, and increased physical activity should be encouraged to prevent, delay, or slow down the progression of MCI [30]. The most compelling data are for the role of exercise in reducing the risk of dementia [32, 33]. Table 1 summarizes a range of interventions that may reduce the risk of MCI and progression to dementia, and indicates, based on the expert opinion of the authors, the strength of the evidence supporting specific recommendations [3, 4, 9, 32, 34–46].

**Table 1:** Lifestyle interventions recommended for MCI based on expert opinion

Intervention	Recommendation	Degree of confidence*
Medical	• Ensure blood pressure is optimal [34]	+++
	• Ensure body mass index (BMI) is optimal [35]	++
	• Ensure cholesterol level is optimal [36]	+
	• Ensure no undiagnosed diabetes or if diabetic ensure control is optimal for age [37]	+
	• Review medicines and assess for anticholinergic burden [38]	+
	• Ensure hearing loss is addressed [3]	+
	• Adopt practices to avoid head injury (use helmets, avoid unprotected contact sports) [39]	
Lifestyle	• Advise smoking reduction and cessation support [40]	+++
	• Advise limiting alcohol intake in line with currently accepted guidelines [41]	++
	• Encourage physical activity and exercise [32, 33, 42]	+++
	• Protect against head injury [39]	+
	• Encourage brain fitness activities and social connectedness	
	• Promote good sleep patterns and adequate sleep time	
Psychosocial	• Adequately treat depression and anxiety [43]	+++
	• Advise on methods of cognitive training [44]	++
	• Recommend opportunities for increasing social engagement [45]	+
	• Reduce stress	
Nutritional	• Advise on dietary principles around maintaining general health [4]	++
	• Encourage adherence to diets with some evidence of benefit, such as Mediterranean, MIND, and DASH [46]	+++
	• Recommend evidence-based nutritional supplements, such as Souvenaid, are considered [9]	+++

*Degree of confidence is based on an expert assessment of published evidence and personal experience, rather than a formal, systematic, evidence-based review: +++, high degree of confidence with strong supporting evidence from randomized controlled trials; ++, good degree of confidence with supporting evidence; +, fair degree of confidence with some supporting evidence

National guidelines provide generally consistent recommendations for the management of MCI [16, 17, 47]. Pharmacologic interventions with AD drugs are generally not recommended for patients with MCI but may be considered if there is biomarker evidence of AD, although this opinion is based on limited clinical trial evidence [48]. Changes to lifestyle and diet are encouraged, with the proviso that benefits may be modest [49]. Epidemiological studies have shown an association between diets with high antioxidant content, such as the Mediterranean diet, and a decreased risk of dementia [50], MCI [51], and cognitive decline [52–56] in older adults. The evidence also suggests that multimodal intervention in lifestyle risk factors is more appropriate than focusing on single parameters [56, 57]; single nutritional supplements are not recommended because of insufficient evidence of clinical benefit [5].

Expert opinion:

- A multimodal approach is recommended; dietary and nutritional interventions should be considered alongside individualized lifestyle modifications.
- Pharmacologic therapy, except for the treatment of depression or other neuropsychiatric symptoms, is usually not appropriate for patients diagnosed with MCI.

## Rationale for nutritional interventions

The association between diet, nutritional status, and healthy brain aging has provided a rationale for the investigation of supplements to improve cognitive function in patients with MCI or AD [4, 58]. Single-agent nutrient supplements tested include vitamin E [59], vitamin C [60], B vitamins [61–63], vitamin D [64], flavonoids [65], carotenoids [65, 66], and omega-3 fatty acids [67]. Based on the existing trials, however, there is insufficient evidence to support the use of single-agent nutrients to modify the course of cognitive decline in patients with MCI [5]. The body of evidence showing the role of dietary and nutritional factors in MCI and AD is constantly evolving and has been reviewed extensively in recent papers [68–70]. Despite the failure of numerous studies to show benefits for single-agent nutrient supplements [5], there are compelling reasons to consider a nutritional approach in conjunction with lifestyle interventions for the management of MCI [71], for example, to enhance the supply of precursors required to make neuronal membranes and synapses [72].

The neuronal membranes are composed of a phospholipid bilayer containing cholesterol and other lipids [73, 74]. Changes in the composition of these lipids and phospholipids are associated with several neurological and psychiatric diseases [75]. There is a growing consensus on changed phospholipid profiles in patients with AD [76], reflecting disturbed phospholipid metabolism [77], which occurs early in the AD process [78, 79]. Phospholipids are affected by several of the lifestyle interventions recommended in the management of MCI, including exercise [80], smoking cessation [81], sleep quality [82], and dietary modification [83]. The reported benefits of combining multimodal lifestyle interventions [56, 57] could be mediated at least in part by cumulative effects on normalizing phospholipid metabolism. By improving phospholipid metabolism, lifestyle interventions may help to preserve neuronal functions and maintain cognitive performance. Evidence suggests that key nutrients required for phospholipid synthesis are lower in the blood and cerebrospinal fluid of patients with MCI [84] and such nutritional deficiencies may impair the brain’s ability to maintain neuronal functions.

The metabolic pathway responsible for producing brain phospholipids can be positively influenced by supplying a combination of nutritional precursors and cofactors (reviewed in Wurtman et al. [85]). Nutrients appear to act synergistically, which suggests that a multinutrient approach could be more effective than supplementation with a single nutrient [71].

Several studies have shown that nutrient intervention can increase plasma levels of nutrients involved in phospholipid synthesis, but most failed to demonstrate clinical benefits [86–96]. More beneficial effects may be achieved by addressing the complete specific nutritional requirement for phospholipid synthesis, including phospholipid precursors, nutritional cofactors, and antioxidants.

Expert opinion:

- Although single-agent nutritional supplements have failed to produce cognitive benefits for patients with MCI, a broader nutritional approach warrants consideration.

## Clinical trials of Souvenaid

Souvenaid is a once-daily drink containing a mixture of precursors and cofactors (long-chain omega-3 fatty acids, uridine, choline, B vitamins, vitamin C, vitamin E, and selenium) necessary for the formation and function of neuronal membranes and synapses [71].

The first clinical trials of Souvenaid (Table 2) showed encouraging effects on memory performance in patients with mild AD dementia. Souvenaid was associated with a statistically significant improvement in memory performance in patients with mild and very mild AD dementia, observed over 12 weeks in Souvenir I and 24 weeks in Souvenir II [6, 7], and patients continued to exhibit improved memory for up to 48 weeks [97]. Another trial in patients with mild-moderate AD dementia showed that Souvenaid could be taken safely with standard AD drugs; however, no significant cognitive improvements were demonstrated [8]. These preliminary data suggested that the benefits of Souvenaid were most likely to be seen at the early end of the AD spectrum, and subsequently, an independent trial group (LipiDiDiet) designed a trial in patients with MCI due to AD (prodromal AD) [9].

**Table 2:** Summary of randomized clinical trials of Souvenaid in patients with prodromal AD (MCI due to AD), mild AD dementia, and mild-moderate AD dementia

Population	Prodromal AD^a^	Mild AD dementia^b^	Mild AD dementia^c^	Mild-moderate AD dementia^d^
Reference	LipiDiDiet [9]	Souvenir II [7, 97]	Souvenir I [6]	S-Connect [8]
AD drug use	No	No	No	Yes
Intervention duration	24 months	24 weeks (+ 24-week extension)	12 weeks (+ 12-week extension)	24 weeks
No. of patients randomized	311	259	225	527
Country	Finland, Germany, The Netherlands, Sweden	The Netherlands, Germany, Belgium, Spain, Italy, France	The Netherlands, Germany, Belgium, UK, USA	USA
Ethnic origin	99% White	Not stated	Not stated	94% White
Mean age (years)	71	73.8	73.7	76.7
Male/female (%)	49.5/50.5	51/49	50/50	48/52
Average MMSE	26.6	25	23.9	19.5
Primary outcomes	NTB composite score measuring cognition	NTB memory domain composite score	WMS-r delayed verbal recall measuring episodic memory Modified 13-item ADAS-cog assessing cognition	11-item ADAS-cog measuring cognition
Secondary outcomes	CDR-SOB Brain volumes based on MRI (3D T1-weighted anatomical scans of total hippocampal, whole brain, and ventricular volumes Progression to dementia Nutritional blood parameters	NTB executive function domain NTB total composite score DAD EEG Nutritional blood parameters	ADCS-ADL WMS-r immediate verbal recall CIBIC-plus NPI QOL-AD Nutritional blood parameters	Cognitive test battery (Digit Span-WMS, concept shifting test, letter digit substitution test, and category fluency) ADCS-ADL CDR-SOB Nutritional blood parameters

*ADAS-cog* Alzheimer’s Disease Assessment Scale-cognitive subscale, *ADCS-ADL* Alzheimer’s Disease Co-operative Study-Activities of Daily Living, *CDR-SOB* Clinical Dementia Rating Sum of Boxes, *CIBIC-plus* Clinician Interview-Based Impression of Change plus Caregiver Input, *CSF* cerebrospinal fluid, *DAD* Disability Assessment for Dementia Scale, *EEG* electroencephalography, *MEG* magnetoencephalography, *MMSE* Mini-Mental State Examination, *MRI* magnetic resonance imaging, *NPI* neuropsychiatric inventory, *NTB* neuropsychological test battery, *QOL-AD* Quality of Life in Alzheimer’s Disease, *WMS-r* Wechsler Memory Scale-revised

^a^Prodromal AD as defined by episodic memory disorder (performance below one standard deviation on two of eight cognitive tests [at least one on memory]) and evidence for underlying AD pathology based on positive findings from at least one of the following diagnostic tests: CSF, MRI, and ^18^F fluorodeoxyglucose (^18^F-FDG) PET analysis

^b^Probable AD according to the National Institute of Neurological and Communicative Disorders and Stroke and the Alzheimer’s Disease and Related Disorders Association (NINCDS-ADRDA) criteria, an MMSE score of ≥ 20, and recent magnetic resonance imaging (MRI) or computed tomography (CT) scan had shown no evidence of any other potential causes of dementia

^c^Probable AD according to the NINCDS-ADRDA criteria, a MMSE score of 20–26, and a recent MRI or CT scan compatible with AD

^d^Probable AD according to the NINCDS-ADRDA criteria, a MMSE score between 14 and 24 inclusive, and use of US Food and Drug Administration-approved AD medication on a stable dose for at least 4 months prior to baseline

In 2009, the European LipiDiDiet Group started a 24-month randomized, controlled, double-blind, parallel-group, multicenter trial in patients with prodromal AD [9]. Participants were randomly assigned (1.1) to active product (125 ml once a day Souvenaid) or control product and were not receiving cholinesterase inhibitors at baseline. The primary endpoint was a change in a neuropsychological test battery (NTB; composite *z*-score based on the Consortium to Establish a Registry for Alzheimer’s Disease [CERAD] 10-word list learning immediate recall, CERAD 10-word delayed recall, CERAD 10-word recognition, category fluency, and letter digit substitution test). Although the intervention had no significant effect on the primary endpoint over 2 years, cognitive decline in this trial population was much lower than expected in both the treatment and the placebo groups; therefore, the primary endpoint was inadequately powered. The LipiDiDiet trial showed significant differences in secondary endpoints. Significant benefits were reported for Souvenaid in the CDR-SOB and ADCOMS [9, 10]. In addition, a per-protocol analysis, which excluded patients with major protocol violations (most commonly, failure to comply with study product intake), showed a benefit in episodic memory (three-item memory composite *z*-score); this was not significant in the intention-to-treat analysis. Brain imaging with MRI showed significant reduction of hippocampal atrophy and less expansion of ventricular volume in patients taking Souvenaid.

Taken together, the 4 randomized controlled trials including a total of 1332 patients with prodromal AD or mild-moderate AD dementia (Table 2) showed that Souvenaid was well tolerated [6–9]. Only 1 of these trials (LipiDiDiet trial) specifically studied the use of Souvenaid in MCI due to AD (prodromal AD). Adverse events reported in the LipiDiDiet trial are shown in Additional file 1: Table S1.

An effect size analysis was done to see whether the effects of Souvenaid are clinically detectable in patients with early AD [98]. Effect sizes > 0.2 are considered large enough to be clinically meaningful [99]. The calculated effect sizes (Cohen’s *d* statistic) were 0.21 (95% confidence intervals − 0.06, 0.49) for the primary outcome in Souvenir II (NTB memory *z*-score) and 0.20 (0.10, 0.34) for the co-primary outcome of Souvenir I (Wechsler Memory Scale delayed recall). The number needed to treat (NNT) values for Souvenaid were 6 for Wechsler Memory Scale-revised (WMS-r) delayed memory (> 0) in Souvenir I, 9 for NTB memory (≥ 0.3), and 21 for NTB memory (≥ 0.0) in Souvenir II. The low NNT and high number needed to harm (NNH) indicated a favorable harm-to-benefit ratio for Souvenaid in patients with mild AD dementia [98]. Additional data from the LipiDiDiet trial showed effect sizes of 0.17 for the primary NTB endpoint and 0.33 for the secondary CDR-SOB endpoint, with NNT values of 10 and 6, respectively, and high NNH values [9]. Effect size analyses help to inform the discussion about whether interventional effects of Souvenaid may be considered clinically meaningful and suggest that benefits are greatest when using Souvenaid early in the course of AD.

A meta-analysis of published clinical trials showed Souvenaid was associated with improvements in verbal recall in patients at early stages of AD dementia. Souvenaid had no detected beneficial effects on functional ability, behavior, or global clinical change over the broad spectrum of AD [100]. However, this meta-analysis was not based on individual patient data and did not include data from the LipiDiDiet trial.

The data from randomized controlled trials corroborate the putative mode of action of Souvenaid, i.e., improving the supply of nutrients required for phospholipid metabolism and to support neuronal structure and function [85]. Measurements of nutritional biomarkers showed increased nutrient levels [9, 97] and phosphatidylcholine-docosahexaenoic acid levels [76] in the bloodstream and increased choline levels and markers of phospholipid synthesis in the brain revealed by magnetic resonance spectroscopy [101]. Furthermore, electroencephalography showed improved functional network connectivity in patients with early AD [102]. An exploratory study, with a small sample size and unbalanced study groups, did not show any treatment effects using the novel technique of magnetoencephalography [103]. Brain imaging did show a reduced brain atrophy rate in patients with prodromal AD, suggesting a potential effect on the disease process [9]. A post hoc analysis of data from the LipiDiDiet trial showed a correlation between the preservation of hippocampal volume and memory and CDR-SB [104]. Changes in biomarkers, particularly in hippocampal volume, support the proposed mode of action of Souvenaid, but the relation between the biomarkers and clinical outcomes remains hypothetical. Currently, there is insufficient evidence to show that nutritional biomarkers could be used to indicate the efficacy of Souvenaid. Further clinical trial evidence may support the hypotheses generated.

Real-world data and patient experience programs have also reported benefits for Souvenaid in patients with cognitive impairment and mild AD, including increased motivation and social engagement, improved energy levels, physical and mental resilience, and improvements in mood, cognition, and memory associated with a return to functional tasks and hobbies [105–110]. One study showed that Souvenaid was effective on behavioral and functional deficits [105], while another reported improvements in depression, anxiety, and apathy [109]. Furthermore, caregivers reported benefits in the Subjective Changing Scale (SCS) in patients with MCI at high risk of progression to AD taking Souvenaid [110]. It is important to note that data from these studies are not as strong as the data from randomized controlled trials.

Expert opinion:

- Evidence from randomized controlled trials suggests that Souvenaid should be considered as a management option for some patients with early AD, including MCI.

## Who may benefit from Souvenaid?

Randomized controlled trials investigated Souvenaid across a spectrum of patients with AD, ranging from prodromal AD to mild-moderate AD dementia, and the data showed that the benefits are greater when the product is used early in the disease course. In the LipidiDiet study, pre-specified subgroup analyses showed the benefits of Souvenaid on cognition, memory, and hippocampal volume were greater among patients with very mild disease (MMSE ≥ 26) [9]. An exploratory analysis of these data showed that the effect of Souvenaid on CDR-SOB increased with higher baseline MMSE scores. In patients with a diagnosis of dementia, randomized controlled trials of Souvenaid showed significant benefits in mild AD [6, 7], but not in drug-treated mild-moderate AD dementia (MMSE 14–24) [8].

The low NNT and excellent NNH values together with high rates of long-term product adherence show that Souvenaid is a viable option for use in early-stage disease, including MCI due to AD. Biomarkers should be used to support the diagnosis of MCI due to AD (prodromal AD) because the only randomized controlled trial data showing clinical benefit were obtained in this population; subjects in the LipiDiDiet trial had to have evidence for the underlying AD pathology based on the positive findings from at least one diagnostic test (CSF, MRI, and ^18^F-FDG PET) [9]. No studies are available on the effects of Souvenaid in MCI patients with a different diagnostic type. At present, there is insufficient evidence to show that biomarkers could be used at an individual level to see if patients are benefiting from Souvenaid.

Expert opinion:

- Souvenaid should be considered as an option for patients with a diagnosis of MCI due to AD pathology (prodromal AD) or mild AD dementia.
- Souvenaid is not recommended for patients with moderate or advanced AD dementia.

## For how long may Souvenaid be taken?

Based on the findings of the LipiDiDiet trial [9], patients with MCI due to AD should expect to take Souvenaid for at least 2 years. They may continue to take Souvenaid every day until the physician determines that there is no evidence of benefit, intolerance develops, or patients progress to moderate AD. The benefit can be assessed at each clinic visit using objective tools to assess cognitive functions and subjective reports from the patient and carers. Such information should be considered in the context of the patient’s clinical history.

Souvenaid is well tolerated; however, any features of poor tolerability by individual patients could disrupt adherence and lead to discontinuation. In the LipiDiDiet trial, reported adverse events such as headache and diarrhea were similar in Souvenaid and control groups, and dropout rates due to adverse events were not significantly different between the groups (6% vs 4%, respectively; *p* = 0.437).

It is important to inform patients about the need to adhere to long-term daily intake because the clinical trial data show that the greatest clinical benefits were seen among patients taking the product per protocol [7]. Patients should also be informed about the financial implications of using Souvenaid, especially when they are self-funding, as most will be.

When starting Souvenaid, patients and their caregivers should be given realistic advice about their expected disease course. The risk of progression to AD dementia is significantly higher among individuals with prodromal AD (IWG-2 criteria) compared with those without prodromal AD (61% vs 22% progression at 3 years; hazard ratio 4.0 [95% confidence intervals 3.0–5.2]) [14]. Furthermore, progression to AD is more likely in patients with prodromal AD than in those with other forms of MCI [111]. The LipiDiDiet trial continues to monitor the progression rates in subjects continuing to take Souvenaid compared with controls; however, currently, there is not enough evidence to conclude that Souvenaid decreases the rate of progression from MCI to dementia.

Patients progressing from MCI to mild AD dementia may continue to benefit from Souvenaid [6, 7]. Continuing Souvenaid is not recommended for patients progressing from early to moderate or severe AD dementia [8].

Expert opinion:

- Patients with MCI should take Souvenaid for 2 years or longer if there is evidence of continuing benefit.
- Souvenaid should be stopped if intolerance develops, the patient is no longer benefitting, or they progress to moderate-severe AD.

## Implications for practice, policy, and/or research

Early identification of individuals at risk of progression from MCI to AD dementia is crucial to facilitate patient management at a time when pathological changes and clinical deficits are not yet severe [20]. Primary care physicians have an important role to play in referring individuals with suspected MCI for specialist assessment. Currently, however, there are no treatment options recommended in national guidelines to slow or reverse the progression of MCI to dementia. Data suggest that management of patients with a diagnosis of MCI requires a multimodal approach involving lifestyle changes to reduce the effects of modifiable risk factors (hearing loss, obesity, hypertension, smoking, depression, physical inactivity, social isolation, and diabetes mellitus) [3] and to promote healthy nutrition [4, 58].

Encouraging patients to adopt a healthy lifestyle and diet to support cognitive function is an important first step in the management of patients with MCI [4, 58]. In addition, patients should be provided with information about the multinutrient product Souvenaid, which may be considered as an option for patients with MCI; however, it is important to make patients aware that clinical trial data were obtained in patients with a diagnosis of MCI with AD pathology.

## Conclusion

The consensus opinion of the expert panel is summarized in Table 3. Additional research is required to refine the identification of patients most likely to benefit from Souvenaid and to assess response and clinical benefit during long-term management.

**Table 3:** Summary of expert opinion on MCI

• Early diagnosis of MCI requires the use of suitable neuropsychological tests combined with a careful clinical history. Physicians should explore the clinical history because it provides important information about the changes in individual patients, which may alert them to the emerging cognitive impairment even when an objective screening test is normal. • A multimodal approach is recommended; dietary and nutritional interventions should be considered alongside individualized lifestyle modifications. Pharmacologic therapy, except for the treatment of depression or other neuropsychiatric symptoms, is usually not appropriate for patients diagnosed with MCI. • Although single-agent nutritional supplements have failed to produce cognitive benefits for patients with MCI, a broader nutritional approach warrants consideration. • Evidence from randomized controlled trials suggests that Souvenaid should be considered as a management option for patients with early AD, including MCI. • Souvenaid should be considered as an option for patients with a diagnosis of MCI due to AD (prodromal AD) or mild AD dementia. Souvenaid is not recommended for patients with moderate or advanced AD dementia • Patients with MCI should take Souvenaid for 2 years or longer if there is evidence of continuing benefit.	

## Additional file

Additional file 1: **Table S1.** LipiDiDiet trial adverse events in participants randomly assigned to Souvenaid or control [9]. (DOCX 13 kb)

## Data Availability

Data sharing is not applicable to this article as no datasets were generated or analyzed during the current study.

## Abbreviations

AD: Alzheimer’s disease
ADAS-cog: Alzheimer’s Disease Assessment Scale-cognitive subscale
ADCOMS: Alzheimer’s Disease Composite Score
ADCS-ADL: Alzheimer’s Disease Co-operative Study-Activities of Daily Living
Aβ: Amyloid beta
CDR-SOB: Clinical Dementia Rating Sum of Boxes
CERAD: Consortium to Establish a Registry for Alzheimer’s Disease
CIBIC-plus: Clinician Interview-Based Impression of Change plus Caregiver Input
CSF: Cerebrospinal fluid
DAD: Disability Assessment for Dementia Scale
EEG: Electroencephalography
MCI: Mild cognitive impairment
MEG: Magnetoencephalography
MMSE: Mini-Mental State Examination
MRI: Magnetic resonance imaging
NNH: Number needed to harm
NNT: Number needed to treat
NPI: Neuropsychiatric inventory
NTB: Neuropsychological test battery
PET: Positron emission tomography
QOL-AD: Quality of Life in Alzheimer’s Disease
WMS-r: Wechsler Memory Scale-revised
