# Supplementary material for: Souvenaid in the management of mild cognitive impairment: an expert consensus opinion
Source: Alzheimers Res Ther. 2019 Aug 17;11:73. doi: 10.1186/s13195-019-0528-6 (PMC6698334; doi:10.1186/s13195-019-0528-6)
Supplement: Supplementary file 1 — Table S1. LipiDiDiet trial adverse events in participants randomly assigned to Souvenaid or control [9]. (DOCX 13 kb) [file 13195_2019_528_MOESM1_ESM.docx]

**Additional file 1: Table S1 LipiDiDiet trial adverse events in participants randomly assigned to Souvenaid or control [9]**

| **Most common adverse events*** | **Souvenaid group (n=152)** | **Control group (n=157)** |
| --- | --- | --- |
| Vertigo  Diarrhea  Cystitis  Nasopharyngitis  Respiratory tract infection  Urinary tract infection  Fall  Arthralgia  Back pain  Headache  Cough | 6 (4%)  7 (5%)  4 (3%)  7 (5%)  7 (5%)  7 (5%)  11 (7%)  4 (3%)  10 (7%)  9 (6%)  2 (1%) | 12 (8%)  14 (9%)  9 (6%)  16 (10%)  9 (6%)  9 (6%)  8 (5%)  9 (6%)  5 (3%)  12 (8%)  10 (6%) |

*Reported by at least 5% of participants in either group.
